# Treatment of Pineal Region Rosette-Forming Glioneuronal Tumors (RGNT)

**DOI:** 10.3390/cancers14194634

**Published:** 2022-09-24

**Authors:** Anna Michel, Thiemo Florin Dinger, Ramazan Jabbarli, Philipp Dammann, Anne-Kathrin Uerschels, Marvin Darkwah Oppong, Neriman Özkan, Andreas Junker, Ulrich Sure, Karsten Henning Wrede

**Affiliations:** 1 Department of Neurosurgery and Spine Surgery, University Hospital Essen, University Duisburg-Essen, Hufelandstraße 55, 45147 Essen, Germany; 2 Center for Translational Neuro- & Behavioral Sciences (C-TNBS), University Duisburg-Essen, 45147 Essen, Germany; 3 German Cancer Consortium (DKTK) Partner Site, University Hospital Essen, 45147 Essen, Germany; 4 Department of Neuropathology, University Hospital Essen, University Duisburg-Essen, 45147 Essen, Germany

**Keywords:** glioneuronal tumor, RGNT, pineal region

## Abstract

**Simple Summary:**

The WHO classification of tumors of the central nervous system described for the first time the extremely rare entity of rosette-forming glioneuronal tumors (RGNT, CNS WHO grade 1) in 2007. Due to the rarity of this entity in the pineal region, no specific therapy guidelines currently exist. With our large cohort of patients treated at a single center (from August 2018–June 2021) and with the already described cases in the literature, we would like to highlight possible therapy and follow-up concepts. After the main symptoms of headache or generalized epileptic seizure, cystic lesions adjacent to the pineal gland and the third ventricle were diagnosed in imaging. None of the patients underwent additional chemotherapy or radiotherapy after gross total (GTR)/subtotal resection or endoscopic biopsy. In cases where surgical resection seems feasible with a reasonable surgical risk, we advocate GTR. Long-term MRI follow-up is essential to detect a slow tumor progression.

**Abstract:**

Background: Rosette-forming glioneuronal tumor (RGNT) is an extremely rare entity described for the first time in the WHO classification of tumors of the central nervous system in 2007. Predominantly, single case reports of RGNT in the pineal region have been published, and specific therapy concepts are pending. Methods: The study group comprised all patients with the RGNT (CNS WHO grade 1) in the pineal region that underwent microsurgical tumor removal in our center (August 2018–June 2021). Surgical strategy, histological findings, and clinical outcome are presented, and the results are evaluated and compared to published case reports. Results: Four male patients aged under 50 years (range between 20 and 48 years) and one female patient, 51 years old, were included in this study. Chronic headaches and generalized epileptic seizures were the main symptoms. Supra-cerebellar infratentorial gross total tumor resection (GTR) was performed in two cases, two patients underwent subtotal tumor resection, and an endoscopic biopsy was performed in case five. Conclusion: In cases where surgical resection seems feasible with a reasonable surgical risk, we advocate GTR. Regular and long-term MRI follow-up is essential to detect a slow tumor progression. The role of additional chemotherapy or radiotherapy remains unclear.

## 1. Introduction

The incidence of pineal region tumors is very low as they account for only 1% of all central nervous system (CNS) tumors in adults and 3-8% in children [1,2,3,4]. Papillary glioneuronal tumors (PGNT) were first described under the entity of dysembryoblastic neuroepithelial tumor (DNET) in 1995 [5]. The first description of rosette-forming glioneuronal tumors (RGNT) as a distinct tumor entity was published in 1998 [6]. In 2002 Komori et al. proposed a detailed histopathological diagnosis for the entity of RGNT. In this report, two RGNT cases in the pineal region were identified [7] but not yet as a distinct entity. This rare entity was initially reported in the fourth ventricle [7]. Among the different localizations, such as the fourth and third ventricles as well as the cerebellar, the pineal region is a rare localization in which only 5.3% of RGNT are described [8,9,10]. It was classified as a mixed tumor with gliovascular pseudopapillary structures combined with neurocytic rosettes and separate astrocytic components [7,11,12]. Since the World Health Organization (WHO) classification of tumors of the central nervous system in 2007 and the revision in 2016, as well as in 2021 RGNT, is recognized as a distinct tumor entity [3,11,13]. So far, only 14 cases of RGNT in the pineal region have been described in adults and five in children after the description as distinct entity [8,9,10,14,15,16,17,18,19,20,21,22,23]. This study aims to present the possible treatment options and to report on the experience with this very rare tumor entity in our center.

## 2. Material and Methods

This study was performed in accordance with the Declaration of Helsinki and was approved by the local ethics committee of the University Hospital Essen.

### 2.1. Patients’ Cohort and Data Analysis

All adult patients with pineal-region RGNT CNS WHO grade 1 who underwent surgery in our institution between August 2018 and June 2021 were included in the analysis. Preoperatively, all cases were discussed in our institutional tumor board, and the indication for surgery was decided on an interdisciplinary basis. Data collected from the electronic patient records comprised: age, gender, radiological parameters (magnetic resonance imaging [MRI], computed tomography [CT], positron emission tomography [PET] CT), follow-up data (MRI and clinical status), and histopathological diagnosis. Statistical analyses were carried out, with Statistical analysis performed using the R and R-Studio software packages (R version 3.6.2, R-Studio version 1.2.5033, Boston, MA, US).

### 2.2. Systematic Review, Search Strategy, and Acquisition of the RGNT Cohort Data

**Figure 1:**
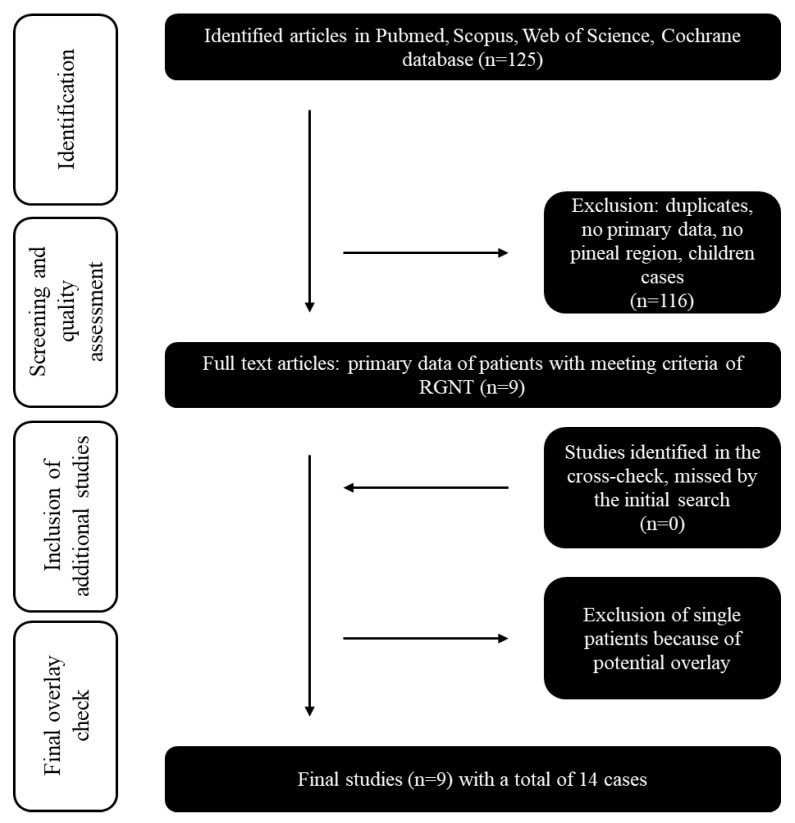
Flow chart of the systematic literature review: Inclusion and exclusion of the initial 125 studies identified by Pubmed, Scopus, Web of Science, and Cochrane databases searches. The year of publication (all cases published before 2007 were excluded) was an exclusion criterion.

The systematic review and meta-analysis were performed according to the PRISMA guidelines [24]. All studies published in English before March 2022 in PubMed, Scopus, Web of Science, and Cochrane Library databases were searched. Only studies that reported pineal-region RGNT CNS WHO grade 1 were included in the final analysis. Reference lists of relevant publications were screened for additional studies. Figure 1 and Supplementary Description S1 visualize the literature selection process. We extracted and summarized all available data from previously reported pineal-region RGNT and our cases based on the selected studies.

## 3. Results

### 3.1. Case Summary

Between August 2018 and June 2021, five patients (aged between 20 and 51 years) with a pineal-region tumor and the histopathological diagnosis of RGNT CNS WHO grade 1 were treated in our center. 

**Figure 2:**
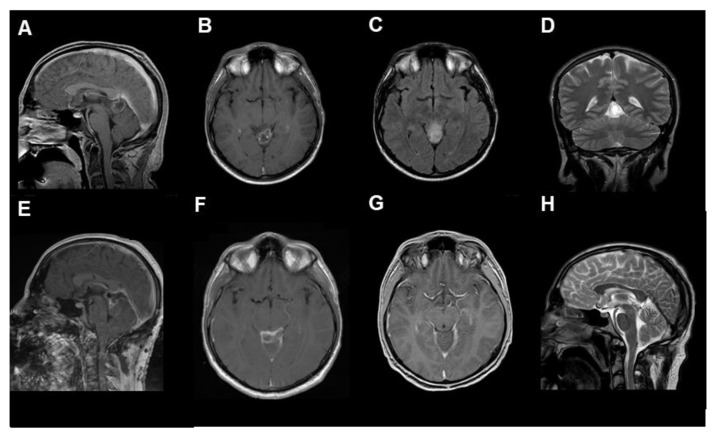
The radiological findings of RGNT in the pineal region ((**A**–**D**) preoperative, (**A**,**B**): T1-weighted with contrast medium, (**C**): T2 flair-weighted, (**D**): T2, (**E**,**F**): postoperative, T1-weighted with contrast medium, (**G**,**H**): 3-months follow-up, (**G**): T1-weighted with contrast medium, (**H**): T2-weighted), from the first case, a 23-year-old male patient who presented with chronic headache.

**Figure 3:**
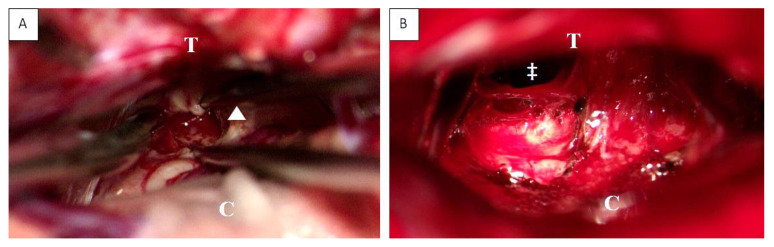
Intraoperative view before (**A**) and after (**B**) supracerebellar, infratentorial tumor resection in the semi-sitting position, exemplarily shown for case 1 (23-year-old male patient, detailed information is presented in Table 1). Abbreviations: T: tentorium; C: cerebellum; double dagger: 3rd ventricle; white arrowhead: tumor.

After contrast-weighted MRI diagnostics (see Figure 2 and Supplementary Figure S1), GTR was performed in two cases. The other two patients underwent subtotal resection. The fifth case underwent an endoscopic biopsy and implantation of a ventriculoperitoneal shunt (Figure 3). In all cases with microsurgical tumor resection, a supra-cerebellar infratentorial approach under continuous electrophysiological monitoring and temporary external ventricular drainage was performed. 

**Figure 4:**
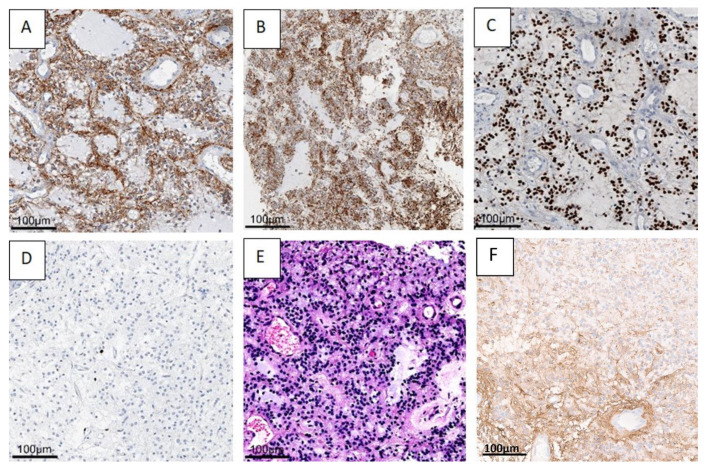
A benign mixed tumor with neurocytic (synaptophysin staining, (**A**) and astrocytic (GFAP) (**F**) staining, olig2 (**C**) staining differentiation. H&E staining (**B**,**E**) shows astrocytic differentiation (**B**), and typical neurocytic rosettes with perivascular pseudorosettes (**E**). In the Ki67 (**D**) staining, only very few proliferation-active cells can be recognized. (Case 1 is presented here). Abbreviations: GFAP—glial fibrillary acidic protein, H&E—haematoxylin and eosin staining.

The histopathologic evaluation of the benign tumor with neurocytic and astrocytic differentiation is shown in Figure 4, using case one as an example.

Case 1:

A 23-year-old male patient presented with chronic headaches for several months. An MRI scan diagnosed a cystic, ring-enhancing lesion (27 × 24 × 23 mm) adjacent to the pineal gland. GTR of the tumor was achieved. After an uneventful postoperative course, the patient could be discharged home without any neurological deficit 10 days after surgery. At 15 months follow-up, there were no new neurological deficits. Due to restricted psychological and mental capacity, the patient was unable to work. Follow-up MRI showed no residual or recurrent tumor.

Case 2:

A 48-year-old male patient presented with a generalized epileptic seizure. The CT and MRI scans demonstrated occluding hydrocephalus with an extensive bithalamic and mesencephal cystic lesion (33 × 30 × 21 mm). An additional preoperative F18-Fluorethyltyrosine [FET] -PET-CT revealed substantial tracer uptake in the tectum but not in the thalami. An endoscopic third ventriculostomy was followed by subtotal tumor resection. Postoperatively, the patient had a transient gait disturbance and vertical gaze palsy. At the last follow-up 2 years after treatment, the gait disturbance had fully recovered, but the vertical gaze palsy had only partially recovered. As preoperatively, he was still unable to work due to his psychological condition with episodes of severe depression. Follow-up MRI showed stable residual bithalamic tumor masses and no recurrence of the resected tumor.

Case 3:

A 27-year-old male patient presented with a generalized epileptic seizure. A ring-enhancing cystic lesion (15 × 17 × 12 mm) in the tectum was delineated on MRI without accompanying hydrocephalus. A subtotal tumor resection was performed, and the patient could be discharged home without neurological deficits 8 days after surgery. Unfortunately, the patient was lost in follow-up.

Case 4:

A 20-year-old male patient presented with headaches for two weeks. MRI revealed occlusive hydrocephalus due to a small (7 × 10 × 4 mm) non-enhancing tumor extending from the pineal gland into the third ventricle. A ventriculoperitoneal shunt system was placed, as the individual anatomy did not allow for endoscopic third ventriculostomy. Follow-up MRI after three and nine months showed tumor growth with localized contrast enhancement. Gross total tumor resection was achieved. At the last follow-up, nine months after treatment, the patient had returned to work and had no general or focal neurological deficit except for some vertigo. Follow-up MRI showed no residual or recurrent tumor.

Case 5:

A 51-year-old female patient presented with a generalized seizure. MRI showed a multifocal lesion adjacent to the tectum extending into the third ventricle and the lateral ventricles. Endoscopic biopsy in an external hospital revealed DNET as the histopathological diagnosis. Until eight years later, yearly performed follow-up MRI showed marginal lesion growth (15 × 12 × 17 mm). At this point, the patient presented with a compensated occlusive hydrocephalus in our center. Endoscopic biopsy with septostomy and placement of a ventriculoperitoneal shunt was performed. Histopathological re-evaluation revealed an RGNT of the pineal gland (CNS WHO grade 1). The patient had no general or focal neurological deficit after surgery and in the 3-month follow-up examination. The 3-month and 10-month follow-up MRI showed stable findings.

### 3.2. Systematic Review of the Literature

Through the systematic review, 14 adult patients’ cases of pineal-region RGNT CNS WHO grade 1 previously published in the literature could be identified [8,14,15,16,17,18]. The mean age at diagnosis was 29.70 years (SD ± 8.61; range: 18–42 years). There was an equal contribution of male (n = 6, 42.86%) and female (n = 6, 42.86%) patients with subtotal STR resection in 35.70% (n = 5) and GTR in 50.00% (n = 7). The longest postoperative clinical course was 20.5 years after GTR. The patient characteristics and treatments are summarized in Table 1.

## 4. Discussion

As RGNT CNS WHO grade 1 in the pineal region is extremely rare, standard treatment and management guidelines are missing. The present observational study, combined with the systematic literature review, aimed to evaluate treatment regimens and neurological outcomes and expand the knowledge beyond the published cases.

Our single-center series presents one of the largest cohorts of adult patients with RGNT (CNS WHO grade 1) in the pineal region (n = 5). Together with the previous 14 cases, 19 patients are now reported. Due to the rarity of the disease, treatment guidelines are pending. A tumor biopsy, partial, and GTR have been reported as treatment options with varying surgical morbidity [8,14,15,16,18,21,25,26]. We focused on RGNT in the pineal region, which is a surgically challenging location. Unlike tumors in only the fourth or third ventricle, a partially different surgical approach and procedure are necessary. In addition to endoscopic resection, GTR can be achieved via a supra-cerebellar infratentorial approach for RGNT in the pineal region [8,18,27]. This approach carries risks, but we could show that resection of an RGNT can achieve a good clinical outcome and may prevent recurrence in the long-term follow-up. GTR was performed without postoperative deficits in 2 cases, with VI left cranial nerves palsy and dysmetria in one patient. Our patients’ cohort demonstrated a good postoperative outcome, and the follow-up examinations were without new general or focal neurological deficits and tumor recurrences in MRI.

Interestingly, DNET was initially diagnosed in our female patient (case 12, see Table 1) as the WHO classification described this entity as DNET before the revision in 2016 and 2021. The recent histopathological evaluation revealed RGNT. This case demonstrated tumor progress seven years after the first biopsy underlining the importance of long-term follow-up for pineal RGNT.

The literature review and our cases suggest that RGNT has a clear male dominance showing first symptoms before the age of fifty. Although very few cases have been described so far, we would advocate GTR in cases where it seems feasible with reasonable surgical risk. Whatever treatment strategy is chosen, regular and long-term MRI follow-up is mandatory.

## 5. Conclusions

Pineal region RGNT is a very rare tumor entity for which standard treatment and management guidelines are pending. In cases where surgical resection seems feasible with a reasonable surgical risk, we advocate GTR. Regular and long-term MRI follow-up is essential to detect a slow tumor progression. The role of additional chemotherapy or radiotherapy remains unclear.

## Figures and Tables

**Table 1:** Summary of demographic and clinical data for patients with pineal RGNT CNS WHO grade 1. Only adult pineal region RGNT published after 2007 have been listed.

ID	Sex	Age (Years)	Medical History/Main Symptoms	TV or VP	STR	GTR	Complication	Last Follow-Up	Ref.	Year
1	m	20	Headache, anisocoria, dysarthria, ataxia	-		x	n.a.	21 months, no recurrence	Marhold et al. [20]	2008
2	m	22	headache, third nerve palsy	-	x		no	n.a.	Ghosal et al. [14]	2010
3	m	29	headache	-		x	n.a.	n.a.	Frydenberg et al. [15]	2010
4	m	39	headache, diplopia	-		x	VI left cranial nerves palsy, dysmetria	42 months, no recurrence	Xu et al. [16]	2012
5	f	41	headache	VP, TV	x		no	n.a.	Sieg et al. [17]	2016
6	f	38	Headache, diplopia, seizure	-	x		n.a.	residual tumor, 36 months	Medhi et al. [10]	2016
7	n.a.	25.9 *	headache	-	n.a.	n.a.	no	n.a.	Yang et al. [8]	2017
8	n.a.	25.9 *	headache	-	n.a.	n.a.	no	n.a.	Yang et al. [8]	2017
9	m	22	headache, diplopia	TV		x	no	n.a.	Muhammad et al. [18]	2020
10	f	30	Headache	VP	x		n.a.	62 months, no recurrence	Lin et al. [19]	2021
11	f	40	Headache, diplopia			x	n.a.	Died 20.5 years later	Lin et al. [19]	2021
12	f	23	Seizure, headache, apasia	VP		x	n.a.	15 months no recurrence	Lin et al. [19]	2021
13	f	42	Ataxia	TV	x		n.a.	14 months no recurrence	Lin et al. [19]	2021
14	m	18	headache	TV		x	n.a.	12 months no recurrence	Lin et al. [19]	2021
15	m	23	headache	-		x	no	15 months no recurrence	current case 1	2022
16	m	48	epileptic seizure, gaze palsy	TV	x		persistent vertical gaze palsy	24 months, no recurrence	current case 2	2022
17	m	27	epileptic seizure	-	x		no	no	current case 3	2022
18	m	20	headache	VP		x	no	15 months, no recurrence	current case 4	2022
19	f	44#	seizure	VP	biopsy		no	9 months, no recurrence	current case 5	2022

Abbreviations: m: male, f: female, STR: subtotal resection, GTR: gross total resection, TV: third ventriculostomy, VP: ventriculo-peritoneal shunt, n.a.: not available, Ref.: references, * mean age, RGNT: rosette-forming glioneuronal tumor of the pineal gland, #initial 44 years old (DNET), then 2021 51 years old.

## Data Availability

The data presented in this study are available on request from the corresponding author. The data are not publicly available due to ethical restrictions.

## Supplementary Materials

The following supporting information can be downloaded at: https://www.mdpi.com/article/10.3390/cancers14194634/s1, Supplementary Description S1: Overview of search terms and operators used for the four different search engines (Pubmed, Scopus, Web of Science, Cochrane Library). Supplementary Figure S1: The key MRI (T2-weighted) photograph of all 5 cases. A-E: Case 1–5.

## Abbreviations

CNS	central nervous system
CT	computed tomography
DNET	dysembryoblastic neuroepithelial tumor
FET	F18-Fluorethyltyrosin
GTR	gross total resection
MRI	magnetic resonance imaging
PGNT	papillary glioneuronal tumors
PET	positron emission tomography
RGNT	rosette-forming glioneuronal tumors
WHO	World Health Organization
