# Supplementary material for: Treatment of Pineal Region Rosette-Forming Glioneuronal Tumors (RGNT)
Source: Cancers (Basel). 2022 Sep 24;14(19):4634. doi: 10.3390/cancers14194634 (PMC9562242; doi:10.3390/cancers14194634)

Supplementary Description S1: Overview of search terms and operators used for the four different search engines (Pubmed, Scopus, Web of Science, Cochrane Library)

1) Pubmed search (February 28th, 2022)

   "(((rosette-forming glioneuronal tumor) AND (pineal)) AND (english[Language])) AND (journal article[Publication Type])"

   Studies identified: 17

2) Scopus search (February 28th, 2022)

   " ALL (rosette-forming AND glioneuronal AND tumor) AND ALL (pineal AND region) AND LANGUAGE (english) "

   Studies identified: 103

3) Web of Science search (February 28th, 2022)

   "ALL FIELDS: (rosette-forming glioneuronal tumor) AND ALL FIELDS: (pineal region) AND LANGUAGE: (English) AND DOCUMENT TYPES: (Article)"

   Studies identified: 5

4) Cochrane Library search (February 28th, 2022)

   "rosette-forming glioneuronal tumor in All Text AND pineal region in All Text - (Word variations have been searched)"

   Studies identified: 1

Supplementary Figure S1: The key MRI (T2-weighted) photograph of all 5 cases. A-E: Case 1-5.

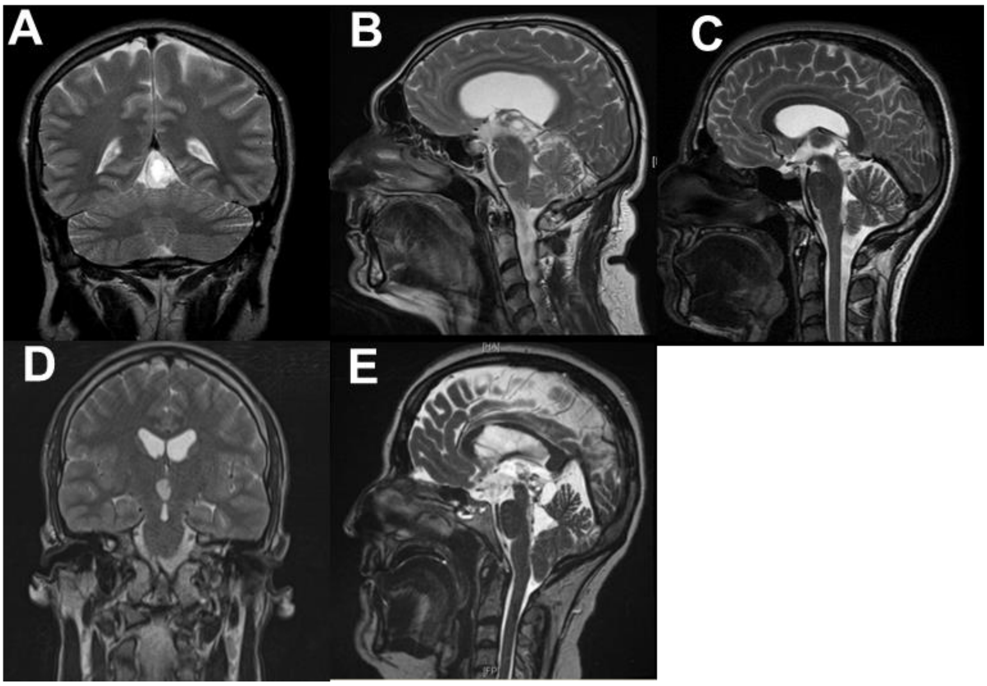

Supplement: Supplementary file 1 [file cancers-14-04634-s001.zip › cancers-1910311-supplementary.pdf]
